# Contractile asymmetry and survival in patients with left bundle branch abnormality treated with cardiac resynchronization therapy

**DOI:** 10.1093/ehjimp/qyad045

**Published:** 2023-12-20

**Authors:** Nareen Kader, Liv Therese Holm-Nielsen, Bhupendar Tayal, Sam Riahi, Anders Sommer, Jens Cosedis Nielsen, Mads Brix Kronborg, Charlotte Stephansen, Niels Holmark Andersen, Niels Risum, Peter Søgaard, Tomas Zaremba

**Affiliations:** Department of Cardiology, Aalborg University Hospital, Hobrovej 18-22, 9100 Aalborg, Denmark; Department of Cardiology, Aalborg University Hospital, Hobrovej 18-22, 9100 Aalborg, Denmark; Department of Cardiology, Aalborg University Hospital, Hobrovej 18-22, 9100 Aalborg, Denmark; Department of Cardiology, Aalborg University Hospital, Hobrovej 18-22, 9100 Aalborg, Denmark; Department of Cardiology, Aalborg University Hospital, Hobrovej 18-22, 9100 Aalborg, Denmark; Department of Cardiology, Aarhus University Hospital, Aarhus, Denmark; Department of Clinical Medicine, Aarhus University, Aarhus, Denmark; Department of Cardiology, Aarhus University Hospital, Aarhus, Denmark; Department of Cardiology, Aarhus University Hospital, Aarhus, Denmark; Department of Cardiology, Aalborg University Hospital, Hobrovej 18-22, 9100 Aalborg, Denmark; Department of Cardiology, Copenhagen University Hospital Rigshospitalet, Copenhagen, Denmark; Department of Cardiology, Aalborg University Hospital, Hobrovej 18-22, 9100 Aalborg, Denmark; Department of Cardiology, Aalborg University Hospital, Hobrovej 18-22, 9100 Aalborg, Denmark

**Keywords:** left bundle branch abnormality, pacemaker, coronary disease, cardiomyopathy, mortality

## Abstract

**Aims:**

Currently, electrical rather than mechanical parameters of delayed left ventricular (LV) activation are used for patient selection for cardiac resynchronization therapy (CRT). However, despite adhering to current guideline-based criteria, about one-third of heart failure (HF) patients fail to derive benefit from CRT. This study sought to investigate the prognostic survival significance of a recently introduced index of contractile asymmetry (ICA) based on the deformation of entire opposing LV walls in the context of selecting patients with HF and left bundle branch abnormality (LBBB) for CRT.

**Methods and results:**

We analysed 367 patients with HF and LBBB undergoing CRT (31.6% females, 69 ± 9 years, ischaemic aetiology in 50.7%, LV ejection fraction 27 ± 6%). ICA was calculated using LV strain rate values from curved anatomical M-mode plots of apical 2D echocardiography images. The predictive value of ICA was assessed using Kaplan–Meier analysis and Cox proportional hazards models. During a median follow-up time of 5.54 years, death or cardiac transplantation occurred in 105 (28.6%) cases. Higher baseline ICA values in all apical views, particularly in the two-chamber view (ICA-2ch), were associated with increased event-free survival, and the unadjusted hazard ratio was 0.28 (95% confidence interval 0.18–0.46). Higher ICA-2ch (>0.319 s^−1^) consistently predicted survival across clinical subgroups and remained significant after covariate adjustment, while the event rate sharply increased in low ICA-2ch cases. Additionally, including ICA-2ch improved the predictive value of the multivariate risk model containing the typical LBBB pattern.

**Conclusion:**

Pre-implant ICA suggests a quantitative prognostic threshold for both long-term survival and adverse outcomes following CRT implantation.

## Graphical Abstract

**Graphical Abstract:**
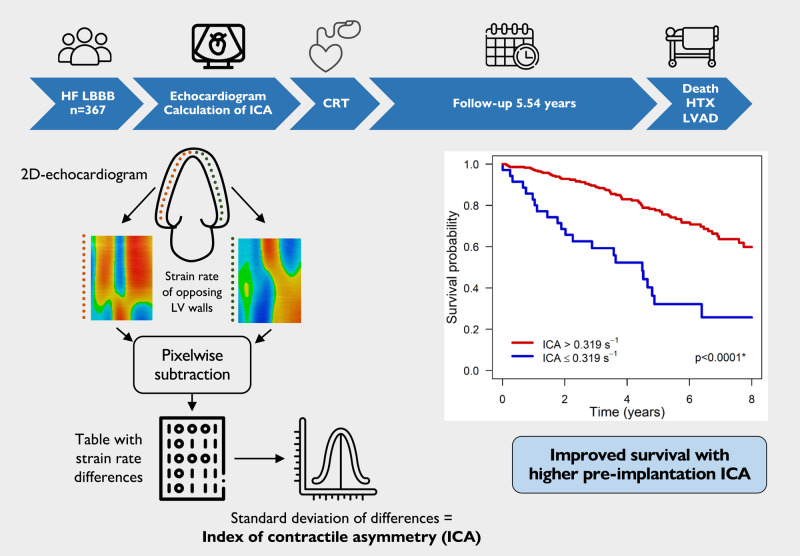

## Introduction

Cardiac resynchronization therapy (CRT) improves morbidity and mortality in patients with electrical conduction abnormalities and heart failure (HF).^1,2^ However, some questions and issues related to CRT including patient selection and outcome prediction remain unresolved.^3^ Besides the presence of symptomatic HF, abnormal electrical activation is the mainstay of patient selection criteria for CRT implantation. Specifically, the left bundle branch abnormality (LBBB) with a QRS duration of ≥150 ms is categorized as a class I indication for CRT, and a QRS duration of 130–149 ms is considered a class IIa indication.^4^ However, many non-responders with LBBB imply that electrical conduction abnormalities are far from sufficient to predict a favourable CRT outcome.^3^ Furthermore, in certain patients within this group, CRT fails to yield the intended disease-modifying effect, leading to a worsening of the condition post-implantation.^5^

In the patient selection for CRT, the role of cardiovascular imaging is limited to the quantification of a left ventricular ejection fraction (LVEF) ≤35% by echocardiography, and the mechanical impairment in terms of contractile dyscoordination is not taken into account. However, several studies have suggested echocardiographic assessment of mechanical LV dyssynchrony to optimize patient selection.^6–9^ These studies imply that mechanical dyssynchrony at baseline is linked to a favourable outcome and hence improved patient selection. This is especially true for dyssynchrony patterns compatible with an underlying correctable conduction disorder such as LBBB.^9,10^

A novel echocardiographic approach, called index of contractile asymmetry (ICA), incorporates the entire opposite myocardial walls instead of a restricted number of data points such as LV strain-based typical LBBB pattern.^6^ This total display of the LV is a quantitative assessment of mechanical dyscoordination, which is associated with a higher degree of reverse LV remodelling subsequent to CRT.^6^ The aim of this study was therefore to investigate the ability of pre-implant ICA to predict long-term prognosis in patients undergoing CRT.

## Methods

### Study subjects

This study was conducted as a retrospective analysis of prospectively collected data from patients with symptomatic HF and LBBB undergoing guideline-based implantation of CRT. The implantations were performed at Aalborg University Hospital (2013–19), Aarhus University Hospital (2011–19), and Gentofte University Hospital (2009–16). The LV lead was placed in the presumed lateral position, and the right ventricular lead was implanted in the septum. Exclusion criteria included permanent atrial fibrillation or flutter, device upgrade, unavailable baseline echocardiography, or baseline echocardiography unsuitable for regular speckle tracking echocardiography (STE)–based strain rate analysis.

The study protocol was approved by the Institutional Review Boards (3-3013-3242/1) at all three centres and complied with the Declaration of Helsinki.

### Clinical characteristics

Medical records of the patients were reviewed to collect the baseline clinical characteristics, including functional class, comorbidities, medical therapy, estimated glomerular filtration rate (eGFR), and QRS duration. All clinical data, electrocardiograms (ECG), and echocardiographic analyses were performed with blinding to the study outcome. Chronic kidney disease (CKD) was defined as an eGFR < 60 mL/min/1.73 m^2^ of body surface area. An ischaemic aetiology of cardiomyopathy was present when at least one of the following requirements were fulfiled: previous diagnosis of an acute coronary event, having undergone a revascularization procedure, or having a significant coronary artery stenosis.^11^

### Outcomes

The combined outcome included death from any cause, heart transplantation, or LV assist device implantation.

### Echocardiographic analysis

STE analysis of LV was performed using 2D pre-implantation echocardiography images on EchoPAC software version 203 (GE Healthcare, Milwaukee, WI). Apical four-chamber, three-chamber, and two-chamber images, acquired at a frame rate of 62.2 ± 10.0 s^−1^, were analysed. LVEF and end-systolic volume (ESV) were estimated using the biplane Simpson’s method by experienced observers. Additionally, baseline echocardiography was analysed regarding the potential presence of typical LBBB pattern as described by Risum *et al.*^12^ Any view with two or more untraceable segments, despite manual correction, was excluded from the STE analysis and ICA calculation. To assess volumetric response to CRT, LVEF and LV ESV were measured in echocardiograms performed 6 months after the CRT implantation.

### Index of contractile asymmetry

**Figure 1:**
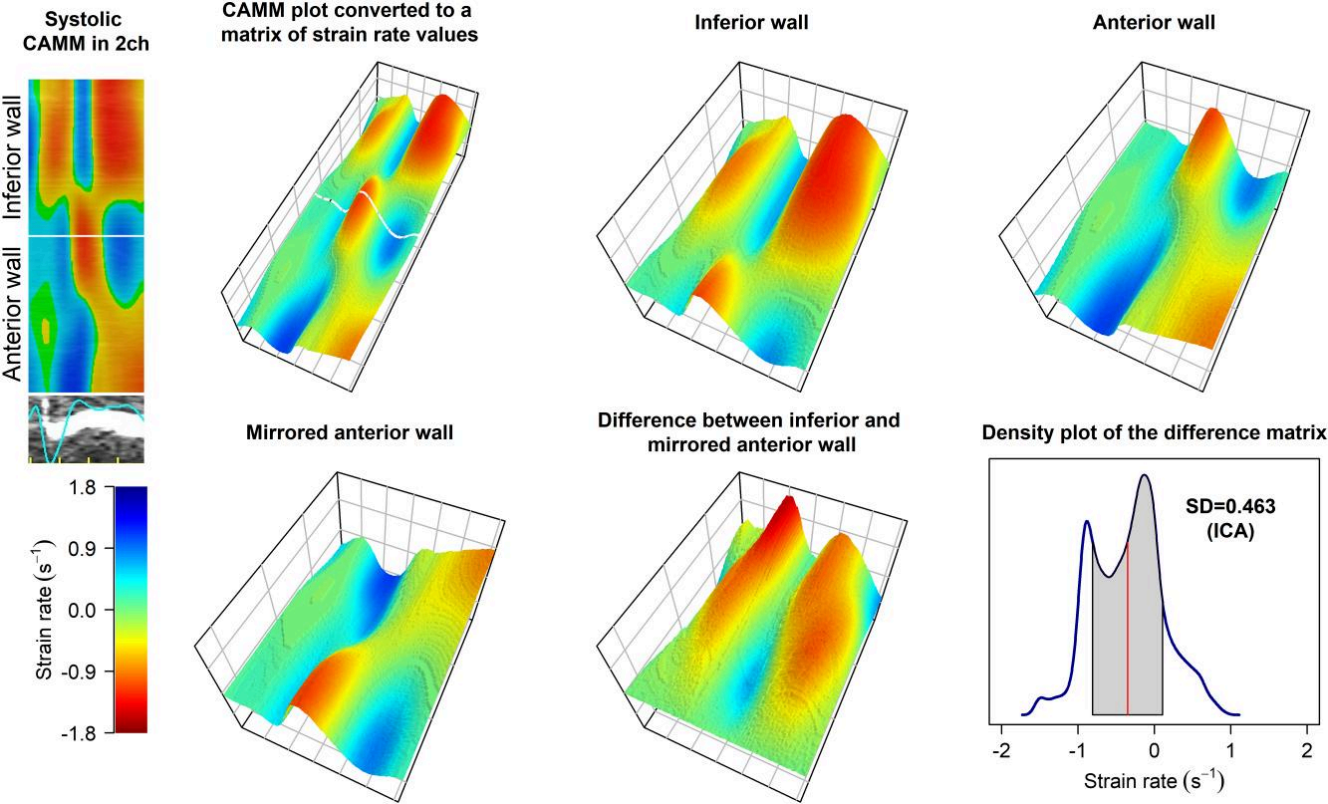
Algorithm of calculation of ICA. An example of two-chamber view in a patient with non-ischaemic cardiomyopathy and typical LBBB pattern. The top left panel shows systolic part of the CAMM plot visible in EchoPAC software. In the following 3D plots, strain rate values are represented using the scale shown in the bottom left panel. Bottom right panel shows a density map of the differences in strain rate values between the opposing walls. SD of the differences (ICA) in this case is 0.463 s^−1^. CAMM, curved anatomical M-mode; SD, standard deviation; ICA, index of contractile asymmetry; LBBB, left bundle branch abnormality.

The definition of systole duration was determined as the period from the onset of the QRS complex to the closure of the aortic valve. The procedures for strain rate analysis and calculation of ICA employed in this study have been previously detailed.^6^ In brief, systolic strain rate data were extracted from the curved anatomical M-mode (CAMM) plots (*Figure 1*). (i) Each pixel in the CAMM plots was transformed into a corresponding strain rate value, forming a matrix (table). Thus, the matrix can be regarded as a structured arrangement where each cell contains a specific strain rate value of the corresponding CAMM plot in pixels. (ii) The matrix was then divided into two equally sized parts representing each opposing LV wall in the specific apical view. (iii) The systolic strain rate values in the two matrix halves were subtracted individually in a symmetrical manner. (iv) Following the symmetrical subtraction, ICA was calculated as the standard deviation (SD) of the strain rate differences between the two opposing LV walls.^6^ ICA analysis was conducted individually for each apical view.

### ECG analysis

Analysis of the baseline ECG included assessment of rhythm, QRS duration, and the presence or absence of Strauss criteria.^13^

### Statistical analysis

Continuous variables were presented as a mean along with their SD or median and inter-quartile range (IQR). The normality of data was assessed through visual examination of histograms and QQ plots. Categorical values were presented as absolute numbers alongside their percentages. The two-sample Student’s *t*-test was used to compare continuous variables. Linear regression and Pearson’s correlation coefficient were employed to assess the association between two linear variables. If necessary for model improvement, logarithmic transformation of the data was performed. Kaplan–Meier analysis and univariate and multivariate Cox proportional hazards (PH) method were applied in survival analysis. Multivariate models were constructed using variables that achieved statistical significance in the univariate analysis. Collinearity among predictor variables was assessed using Pearson’s correlation coefficient to evaluate linear relationships and to identify potential impacts on model robustness. Hazard ratios (HRs) with their 95% confidence intervals (CIs) were reported. ICA was dichotomized using the cut-point value corresponding to the strongest correlation with survival, determined using maximally selected rank statistics. To ensure result consistency, the predictive value of ICA was also assessed within clinical subgroups. Additionally, a multivariate restricted cubic spline (RCS) model was applied to examine the quantitative association between ICA and HR for events after correction for other variables associated with outcome after CRT. The performance of 3-year 25% risk predictive models containing variables derived from univariate Cox PH analysis was compared before and after inclusion of ICA using C-statistic, net reclassification index (NRI), and integrated discrimination index (IDI). Intra- and inter-rater reproducibility analysis of ICA was performed using the Bland–Altman method. Two-sided tests were utilized. A *P* < 0.05 was considered statistically significant. The analyses were conducted using R statistical software, version 3.6.1.

## Results

### Demographic characteristics

A total of 367 patients were included in this study (Aalborg *n* = 153, Aarhus *n* = 176, Gentofte *n* = 38). Among them, 99 patients (27.0%) received a CRT-pacemaker, and 268 patients (73.0%) received a CRT-defibrillator.

Patient baseline characteristics are displayed in *Table 1*. The mean age was 69 ± 9 years, with 251 (68.4%) patients being male. Baseline LVEF was 27 ± 6%, and QRS duration was 164 ± 20 ms.

**Table 1:** Baseline characteristics

	*n* = 367
Age, years	69 ± 9
Male sex, *n* (%)	251 (68.4)
Ischaemic aetiology, *n* (%)	186 (50.7)
CKD, *n* (%)	142 (38.8)
NYHA class	
I, *n* (%)	5 (1.4)
II, *n* (%)	180 (50)
III, *n* (%)	167 (46.4)
IV, *n* (%)	8 (2.2)
ACEI/ARB, *n* (%)	344 (93.7)
Beta-blockers, *n* (%)	347 (94.6)
Loop diuretics, *n* (%)	245 (66.8)
Aldosterone antagonists, *n* (%)	219 (59.7)
Statins, *n* (%)	198 (54)
QRS duration, ms	164 ± 20
Strauss criteria, *n* (%)	331 (91.4)
LV ESV, mL	153 ± 62
LVEF, %	27 ± 6
Typical LBBB pattern, *n* (%)	294 (81.2)

ACEI, angiotensin converting enzyme inhibitors; ARB, angiotensin II receptor blockers; CKD, chronic kidney disease (defined as eGFR <60 mL/min/1.73 m^2^); EF, ejection fraction; ESV, end-systolic volume; LBBB, left bundle branch abnormality; LV, left ventricular; NYHA, New York Heart Association.

Strauss ECG criteria for LBBB were met in 331 (91.4%) of the patients. Typical LBBB contraction pattern by echocardiography was present in 294 (81.2%) cases.

### ICA and survival

**Figure 2:**
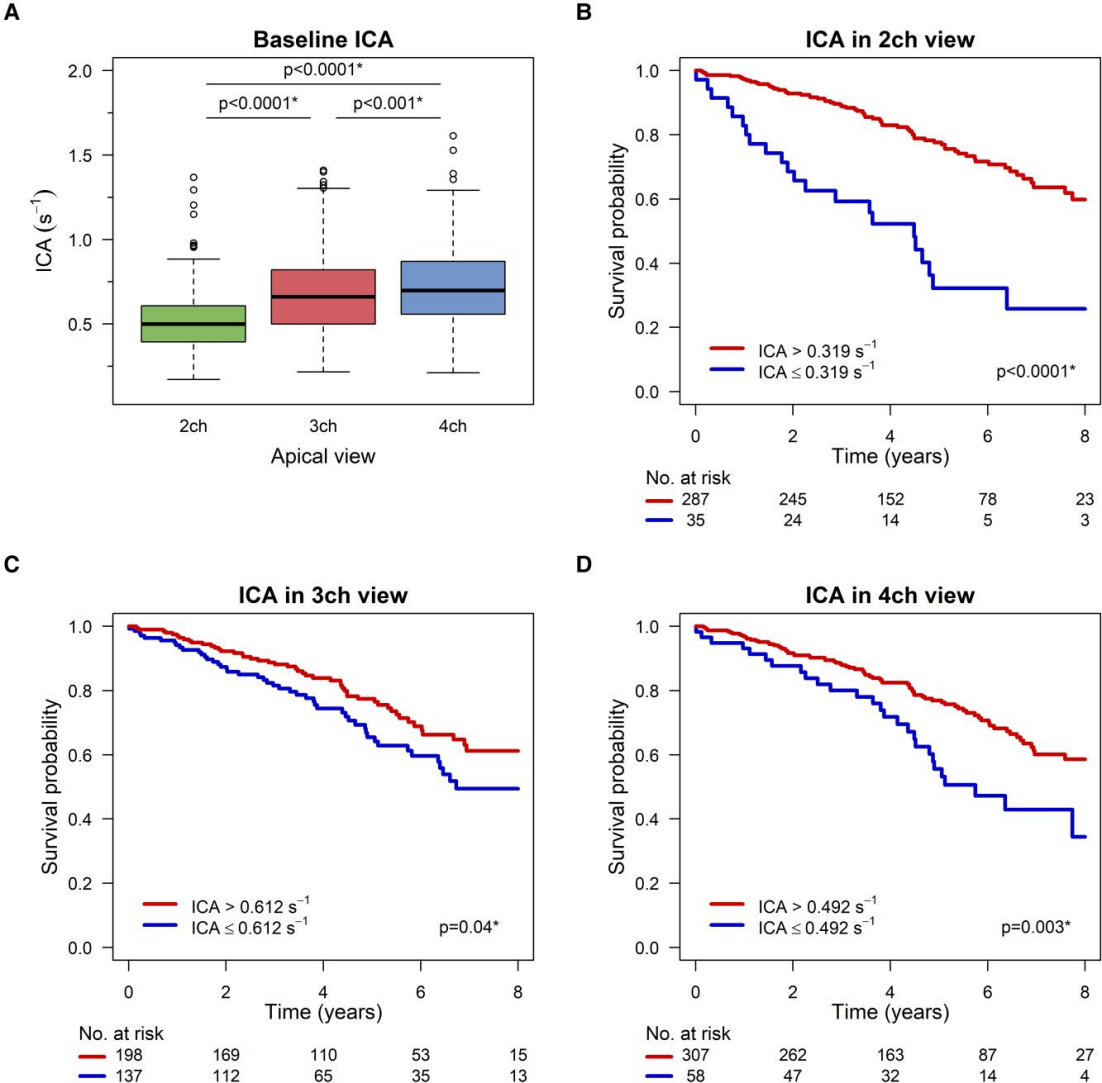
Index of contractile asymmetry and survival after CRT. (*A*) Boxplots of baseline ICA in all three apical echocardiographic views. (*B–D*) Kaplan–Meier curves for event-free survival based on ICA. High and low ICA values are represented by separate curves. ICA, index of contractile asymmetry. *P < 0.05.

*Figure 2A* displays the baseline ICA in the apical echocardiographic views. ICA was 0.519 ± 0.178 s^−1^, 0.687 ± 0.244 s^−1^, and 0.722 ± 0.234 s^−1^ for the two-chamber, three-chamber, and four-chamber views, respectively (all with *P* < 0.001).

The median follow-up time was 5.54 (IQR 3.39–7.3) years, with no loss to follow-up. During this period, 105 (28.6%) patients suffered study events. Among these cases, 102 (97.1%) resulted in all-cause death, while 3 (2.9%) cases involved patients undergoing heart transplantation.

*Figure 2B–D* depict the overall survival probability over an 8-year period, stratified by baseline ICA in all three apical views. Each figure displays event-free survival probability with a high ICA value and survival probability with a low ICA value. In all three apical views, a higher ICA value was associated with significantly higher event-free survival (*P* < 0.0001 in two-chamber view, *P* = 0.04 in three-chamber view, and *P* = 0.003 in four-chamber view).

A stratified survival analysis was conducted based on non-ischaemic vs. ischaemic cardiomyopathy (see Supplementary data online, *Supplement S1*). Elevated ICA-2ch remained significantly associated with improved survival in both patient categories (*P* < 0.001 for both). In non-ischaemic cardiomyopathy, ICA-4ch also remained statistically significantly associated with outcome events (*P* = 0.01).

### Univariate survival analysis

**Figure 3:**
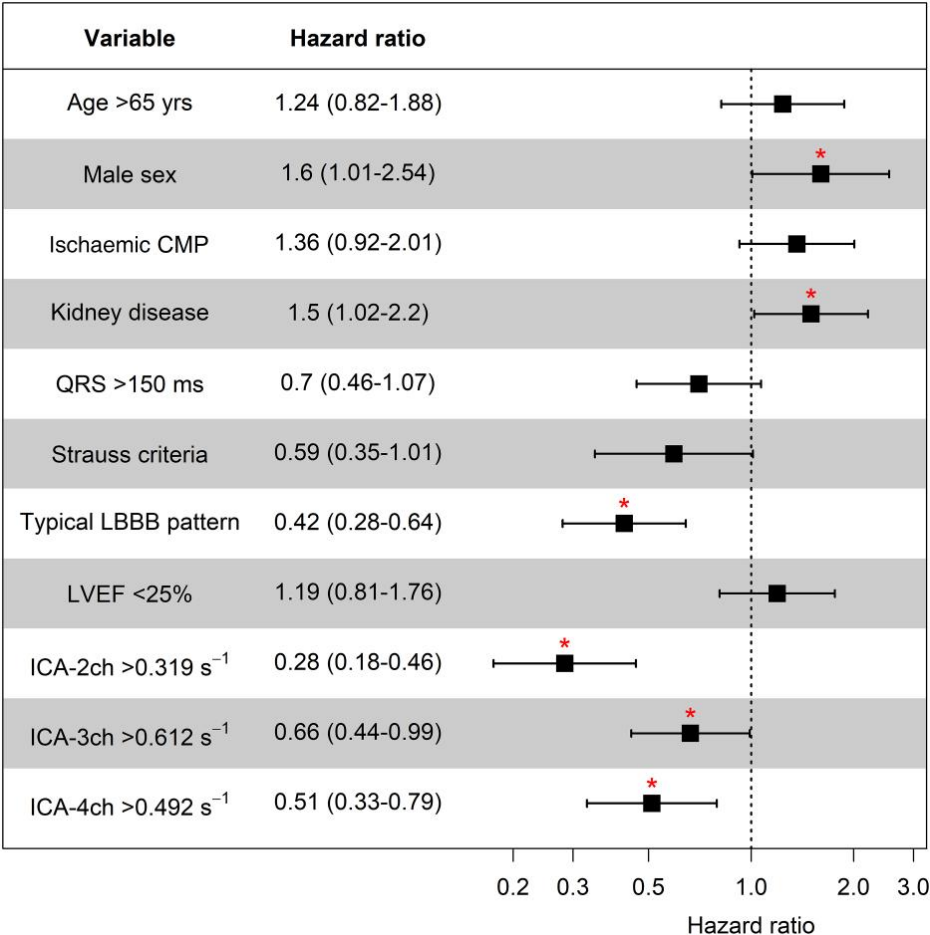
Univariate survival analysis. CKD, chronic kidney disease; CMP, cardiomyopathy; ICA, index of contractile asymmetry; LBBB, left bundle branch abnormality; LVEF, left ventricular ejection fraction. *P < 0.05.

Univariate Cox regression HRs are shown in *Figure 3*. A significantly higher risk of event was observed in males compared with females [HR 1.6 (95% CI 1.01–2.54)] and in patients with CKD [HR 1.5 (95% CI 1.02–2.2)]. Patients exhibiting typical LBBB pattern had a lower risk [HR 0.42 (95% CI 0.28–0.64)].

*Figure 3* also emphasizes the findings presented in *Figure 2B–D*, demonstrating a significant inverse relationship between high ICA values in all apical views and the risk of events. Subjects with an ICA-2ch value above the cut-point of 0.319 s^−1^ [observed in *n* = 287 (89.1%)] had a significantly reduced hazard of events [HR 0.28 (95% CI 0.18–0.46)]. Corresponding patterns were detected in three-chamber view and four-chamber view. In case of ICA-3ch above the cut-point of 0.612 s^−1^ [*n* = 198 (59.1%)], the HR was 0.66 (95% CI 0.44–0.99). Similarly, a significantly lower risk of outcome events was found in the ICA-4ch group above the cut-point of 0.492 s^−1^ [*n* = 307 (84.1%), HR 0.51 (95% CI 0.33–0.79)].

In non-ischaemic cardiomyopathy, HR for ICA-2ch, ICA-3ch, and ICA-4ch was 0.25 (95% CI 0.12–0.52), 0.73 (95% CI 0.39–1.38), and 0.36 (95% CI 0.16–0.82), respectively (see Supplementary data online, *Supplement S1*). In ischaemic cardiomyopathy, the corresponding HRs were 0.32 (95% CI 0.16–0.60), 0.67 (95% CI 0.40–01.14), and 0.61 (95% CI 0.35–1.03).

Three-year event rates, stratified by ICA in the three apical views, are shown in Supplementary data online, *Supplement S2*.

### Multivariate survival analysis

**Figure 4:**
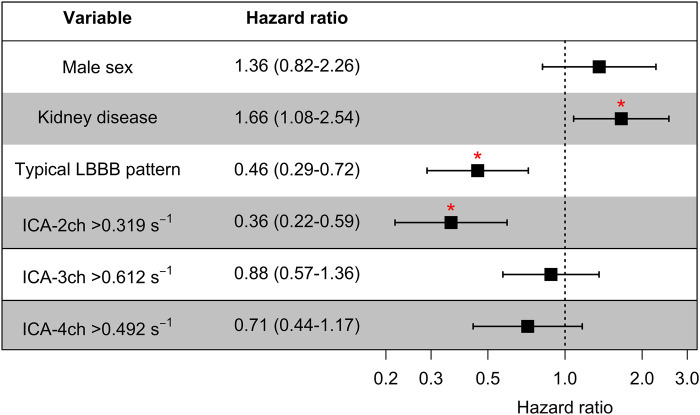
Multivariate survival analysis. The variables, in addition to ICA-2ch, incorporated into the multivariate model are presented. Consistently, the identical set of variables was employed in models where ICA-2ch was substituted with ICA-3ch and ICA-4ch. ICA, index of contractile asymmetry. *P < 0.05.

Sex, CKD, and typical LBBB pattern were included in the multivariate model along with ICA individually in each apical view (see *Figure 4*). Higher values of ICA-2ch maintained a statistically significant and independent association with an improved survival rate, with an HR of 0.36 (95% CI 0.22–0.59). For all pairs of predictor variables in the multivariate models, Pearson’s correlation coefficient ranged from 0.01 to 0.32, indicating a lack of substantial collinearity that could impact the integrity of the models.

### Subgroup analysis

**Figure 5:**
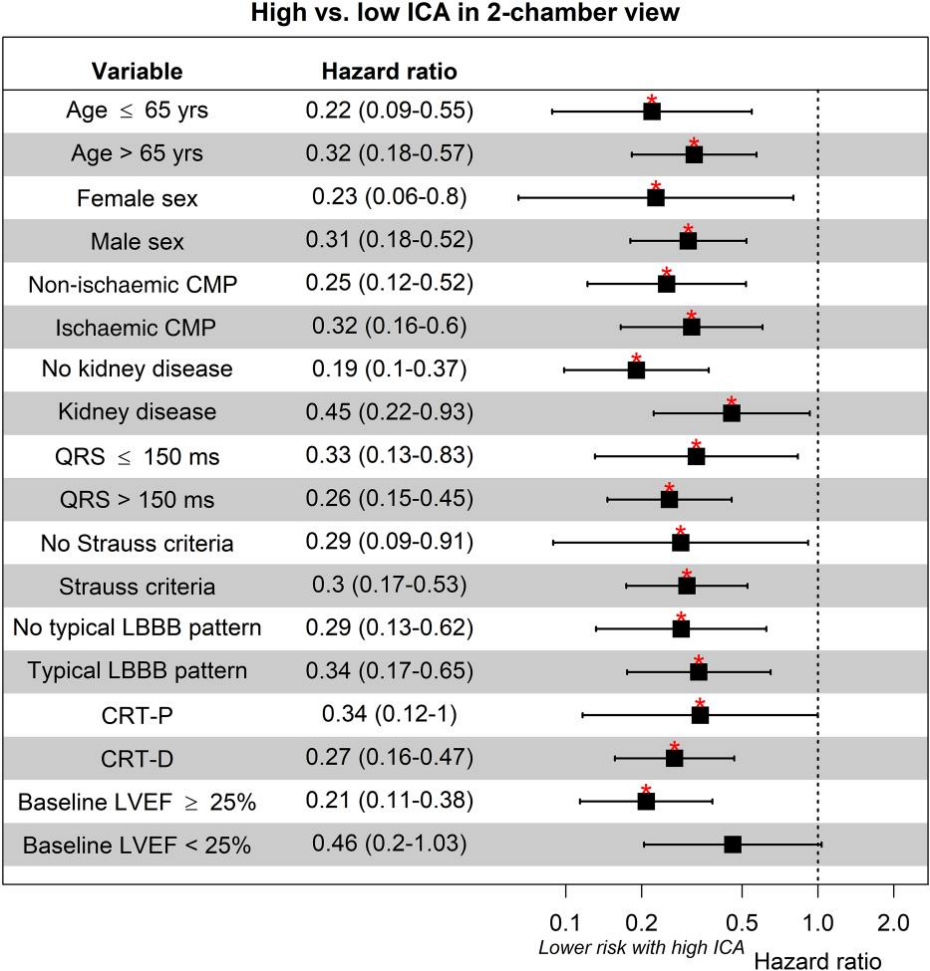
ICA in two-chamber view and survival in clinical subgroups. CMP, cardiomyopathy; CRT-D, cardiac resynchronization therapy defibrillator; CRT-P, cardiac resynchronization therapy pacemaker; ICA, index of contractile asymmetry; LBBB, left bundle branch abnormality; LVEF, left ventricular ejection fraction. *P < 0.05.

The survival analysis of ICA-2ch stratified by clinical subgroups is presented in *Figure 5*. Across all categories, higher ICA-2ch values correlated with improved survival. This observation held also true for the stratification by the presence of Strauss criteria and typical LBBB pattern. Only in the case of LVEF <25%, statistical significance was not reached.

### Restricted cubic splines

**Figure 6:**
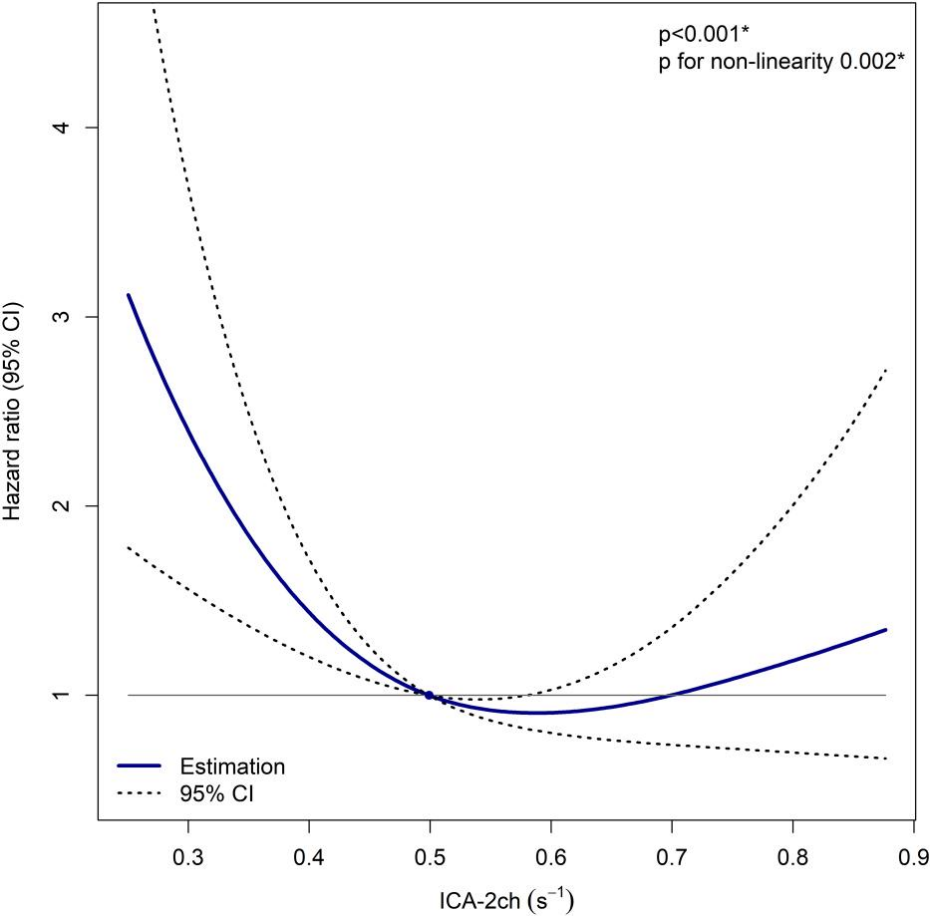
RCS analysis of ICA in two-chamber view and HR for event. The model is corrected for sex, CKD, and typical LBBB pattern. A cut-off at median of ICA in two-chamber view is used (0.499 s^−1^). CI, confidence interval; ICA-2ch, index of contractile asymmetry in two-chamber view. *P < 0.05.

RCS analysis of ICA-2ch, adjusted for sex, CKD, and typical LBBB pattern, is illustrated in *Figure 6*. A statistically significant association between ICA-2ch and HR for event was observed (*P* < 0.001). The HR increased at lower ICA-2ch values while maintaining a relative consistency at higher ICA-2ch values (*P* for non-linearity 0.002).

### Model improvement

A predictive model was built, comprising sex, CKD, and typical LBBB pattern. The C-statistic of the model was 0.66 (95% CI 0.59–0.73). After inclusion of ICA-2ch in the model, C-statistic increased to 0.70 (95% CI 0.63–0.77, *P* = 0.04 for difference). Categorical NRI was 0.18 (95% CI 0.06–0.29, *P* = 0.002), and IDI was 0.05 (95% CI 0.02–0.08, *P* = 0.003). In the case of absent event, 67 (46.9%) subjects were correctly reclassified from high to low risk by the new model. When event was present, the new model correctly reclassified one (10.0%) case from low to high risk and incorrectly reclassified 18 (23.1%) cases from high to low risk.

### LV functional response

The follow-up LV function post-CRT was assessed in 342 (93.2%) cases. A linear correlation was observed between the degree for LVEF change after CRT and baseline ICA in all apical projections: ICA-2ch (*r* = 0.22, *P* < 0.001), ICA-3ch (*r* = 0.37, *P* < 0.0001), and ICA-4ch (*r* = 0.41, *P* < 0.0001; see Supplementary data online, *Supplement S3*). An inverse linear relationship was present between the degree of LV ESV change and pre-implantation values of ICA-3ch (*r* = 0.37, *P* < 0.0001) and ICA-4ch (*r* = 0.38, *P* < 0.0001, respectively). No significant association was reached for LV ESV reduction and baseline ICA-2ch (*r* = 0.09, *P* = 0.11).

### Reproducibility analysis

In both intra-rater and inter-rater ICA reproducibility analyses, 20 randomly selected cases were used. Good reproducibility was observed, with an intra-rater bias of −0.013 (95% CI −0.026 to 0.001) s^−1^, limits of agreement ranging from −0.113 to 0.088 s^−1^, and coefficient of variation of 8.2%. In the inter-rater analysis, the bias was 0.004 (95% CI −0.013 to 0.022) s^−1^, limits of agreement spanning from −0.120 to 0.129 s^−1^, and a coefficient of variation of 10%.

## Discussion

In this analysis of prospectively collected data, the presence of a high ICA was associated with improved event-free outcomes after CRT. The correlation between a higher degree of contractile asymmetry and more favourable prognosis was particularly pronounced in the apical two-chamber view. ICA remained a significant predictor even after stratification by clinical subgroups, including distinctions between ischaemic vs. non-ischaemic cardiomyopathy as well as QRS duration. It is worth noting that traditional predictive LBBB criteria, such as QRS duration and Strauss’ criteria, did not significantly correlate with mortality in this study. This is likely related to the strictly selected LBBB population, underscoring the strength and specificity of ICA.

The quantitative nature of ICA facilitated the identification of a cut-off point, enabling patient stratification based on outcomes. Overall, high ICA levels were not only linked to short-term reverse LV remodelling post-CRT but also served as a predictor for a favourable long-term prognosis, especially when considering high pre-implantation ICA values in two-chamber view. Conversely, low ICA-2ch values were associated with a marked increase in mortality following CRT. This implies the existence of a distinct subgroup of patients who may be adversely affected by CRT, warranting caution in its application to this specific cohort. This also aligns with prior data suggesting that a low degree of pre-implant mechanical dyssynchrony correlates with poor outcome.^14,15^

ICA is a quantitative parameter similar to cross-correlation analysis.^14,16^ However, unlike previous techniques based on tissue Doppler imaging and conventional STE,^12,16–20^ ICA is not confined to analysing a limited number of curves for assessing mechanical dyssynchrony.^6^ This characteristic allows ICA to precisely localize and measure dyssynchrony linked to LBBB in a novel way, potentially optimizing pre-implant CRT selection.

Other studies have also used methods beyond ECG to predict pre- and post-implant response to CRT. These investigations utilize typical LBBB pattern and other LBBB-specific traits, i.e. septal flash and apical rocking, to find a convincing prognostic value both pre- and post-implant.^9,12^ Nevertheless, these approaches are still limited by a restricted myocardial range of view.

Myocardial work, as evaluated through pressure–strain loops incorporating LV pressure, has demonstrated considerable promise in predicting CRT response.^19,20^ This methodology integrates regional strain rate values (the time derivative of strain) in its computations. The alignment of the utilization of strain rate in both ICA and myocardial work introduces a compelling dimension to our comprehension of LV dyssynchrony. Unfortunately, our study did not incorporate blood pressure measurements during echocardiograms, rendering the computation of myocardial work impractical within our data set.

Remarkably, the findings of this study reveal that ICA in two-chamber view was superior to the well-investigated four-chamber and three-chamber views to predict the overall survival probability over time. ICA values in the three-chamber and four-chamber views in the present cohort are higher than those in the two-chamber view, consistent with the LV activation direction in LBBB. Both prior research and this study demonstrate a connection between asymmetry of contraction, as assessed by ICA in the septal–lateral direction, and LV remodelling after CRT. The pre-implantation ICA values can even prognosticate the degree of LV ESV reduction after CRT. Moreover, the reduction of LV ESV itself is quantitatively linked to the decrease in ICA after CRT.^6^

In the univariate analysis of this study, higher baseline ICA in both the three-chamber and four-chamber views correlated with long-term survival. However, ICA in the two-chamber view exhibited an even stronger prognostic value. This can be attributed to a broader expanse of mechanical dyssynchrony, which consequently covers a larger myocardial area and is discernible in two-chamber view. As a result, it provides an expanded sweet spot for optimal LV electrode placement. Theoretically, this broader sweet spot permits greater flexibility in lead positioning, which could be advantageous in clinical scenarios. Notably, the two-chamber view encompasses a substantial portion of myocardial mass that could potentially be engaged after resynchronization. Consequently, achieving improved synchrony in this area could significantly contribute to enhancing overall myocardial performance during CRT.

LBBB’s potential impact on coronary perfusion and its relevance to ICA-2ch’s predictive value is an alternative mechanism worth considering. Skalidis *et al.*^21^ found impaired left anterior descending artery (LAD) blood flow during LBBB, with delayed peak velocity and lower coronary flow reserve in patients with exercise-induced perfusion defects. Youn *et al.*^22^ showed shorter diastolic flow durations in LBBB patients in LAD, especially compared with right ventricular pacing, suggesting a mechanical rather than electrical influence. Our study supports this, favouring the two-chamber view for predicting overall survival due to its reflection of the LAD-supplied myocardial area.

Previous research links positive CRT response to improved LAD flow, while flow in other coronary arteries remains unchanged.^23,24^ Limited studies suggest myocardial ischaemia may increase LV dyssynchrony, as observed by Chen *et al.*^25^ using single-photon emission computed tomography. Thus, LBBB-induced hypoperfusion in LAD may independently contribute to contractile dyscoordination.

Overall, ICA in two-chamber view displayed the strongest association with mortality outcome in this study, despite being less strongly correlated with reverse LV remodelling after CRT compared with the three- and four-chamber views.^6^ A possible explanation for this variance can also be related to different follow-up times, as functional improvement was assessed after 6 months, while follow-up time in the present study was relatively long.

### Future implications

ICA allows an objective and precise quantification of LBBB-associated dyssynchrony and is strongly associated with mortality. However, to gain deeper insights, additional prospective studies are warranted. ICA combined with distinct stages of LBBB delineated by septal deformation, as recently described by Calle *et al.*,^26,27^ warrants a specific consideration in future research. Interaction between ICA and optimal position of CRT leads should be elucidated as well. Preferably, dynamic changes and prognostic implications of ICA after CRT implantation should also be considered.

### Strengths and limitations

One of the strengths of the study is the fact that the ICA method is not subjected to individual assessment. The magnitude of dyssynchrony measured by ICA is calculated automatically from standard STE-based CAMM plots. The operator-independent quantitative assessment allows the method to be automated in a long-term perspective.^6^ Another strength is that echocardiography analyses were conducted independently of end points. Also, only hard end points were taken into consideration, and the study was conducted as a multicentre study.

A limitation of this work is being a retrospective study without a control group. Moreover, the subjects were included on a clinical indication constituting a subjective assessment. Beyond contractile asymmetry, outcome after CRT is multifactorial and depends on patient selection, exact lead position, post-implant medical therapy, optimal device programming, and disease progression.^5^ In terms of electrocardiographic measures of dyssynchrony, only QRS duration and Strauss criteria were used in the study. Although ICA was determined as the SD of the strain rate differences, a normal distribution of the differences could not be ensured.

## Conclusion

In the present observational study, ICA provides an objective echocardiographic cut-off for benefit from CRT. Therefore, it holds promise as a valuable tool for predicting long-term survival prior to CRT implantation, even among patients with a presumed high response rate.

## Supplementary Material

qyad045_Supplementary_Data

## Data Availability

The data underlying this article will be shared upon reasonable request to the corresponding author.

## Lead author biography

Nareen Kader is currently serving in her residency at Department of Cardiology at Nordsjællands Hospital with a completion of her undergraduate and master's degree at Aalborg University, Denmark. At the moment, she is involved in a multicenter clinical study focused on LBBB-related heart failure and examining non-responders to CRT treatment with a novel cardiac imaging approach.
