# Supplementary material for: Contractile asymmetry and survival in patients with left bundle branch abnormality treated with cardiac resynchronization therapy
Source: Eur Heart J Imaging Methods Pract. 2023 Dec 20;1(2):qyad045. doi: 10.1093/ehjimp/qyad045 (PMC11195769; doi:10.1093/ehjimp/qyad045)
Supplement: qyad045_Supplementary_Data [file qyad045_Supplementary_Data.zip › Supplement 2 3-year event rates.docx]

**Supplement 2**

**Three-year event rates stratified by baseline ICA in the three apical views.**


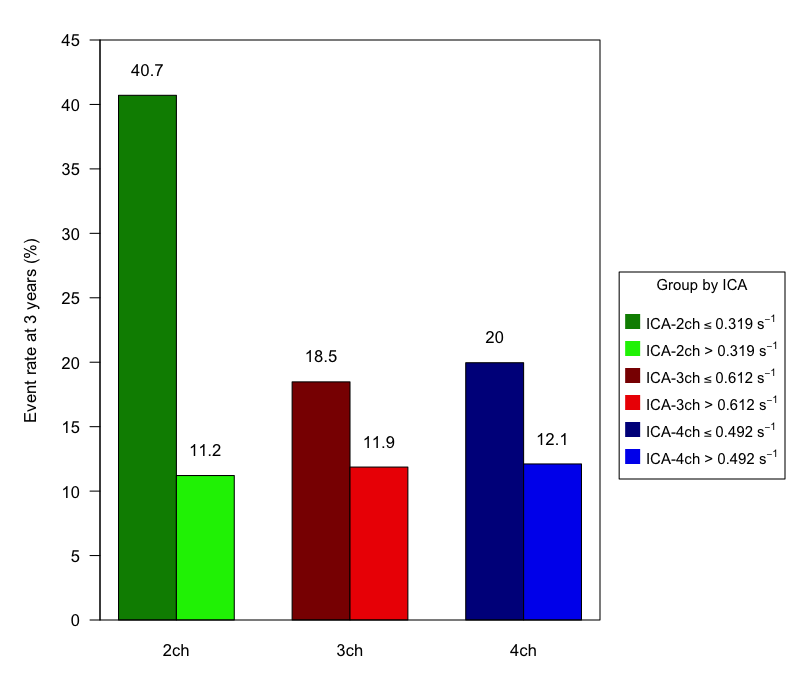


ICA: Index of contractile asymmetry.
