# Supplementary material for: Contractile asymmetry and survival in patients with left bundle branch abnormality treated with cardiac resynchronization therapy
Source: Eur Heart J Imaging Methods Pract. 2023 Dec 20;1(2):qyad045. doi: 10.1093/ehjimp/qyad045 (PMC11195769; doi:10.1093/ehjimp/qyad045)
Supplement: qyad045_Supplementary_Data [file qyad045_Supplementary_Data.zip › Supplement 3 EF ESV.docx]

**Supplement 3**

**Index of contractile asymmetry at baseline and change of left ventricular ejection fraction after six months.**


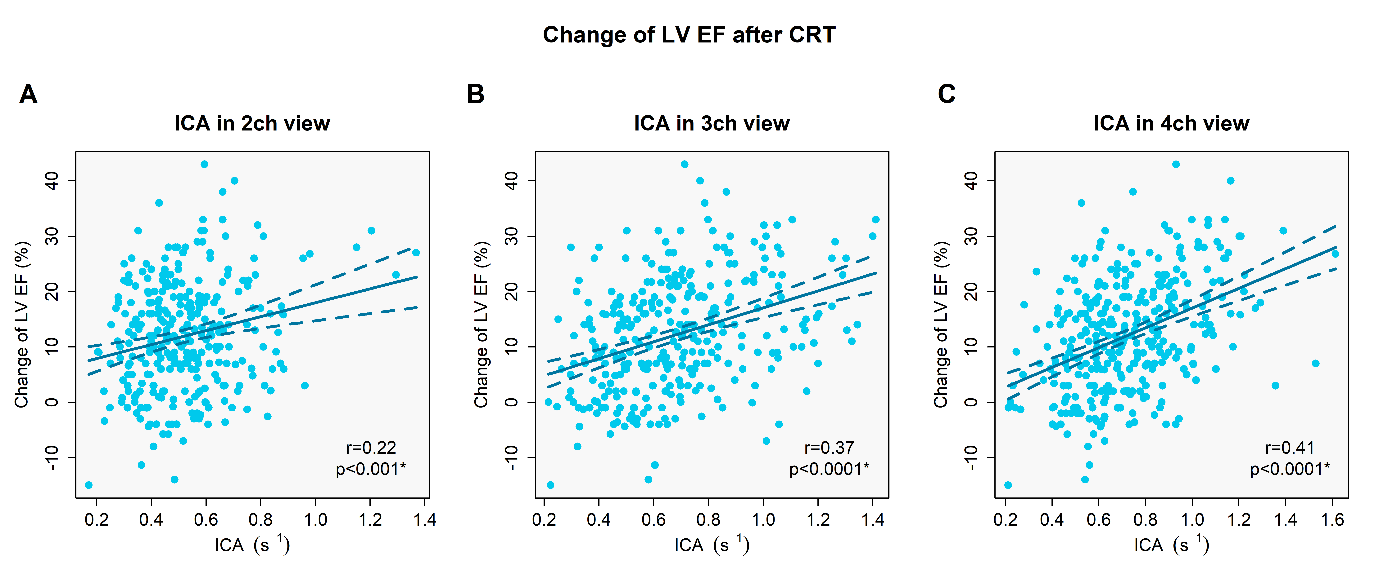


Panels A-C show change of LV EF after CRT as a function of baseline ICA in 2ch, 3ch, and 4ch views, respectively.

CRT: cardiac resynchronization therapy; EF: ejection fraction; ICA: Index of contractile asymmetry; LV: left ventricle; *: p<0.05.

**Index of contractile asymmetry at baseline and change of left ventricular end-systolic volume after six months.**


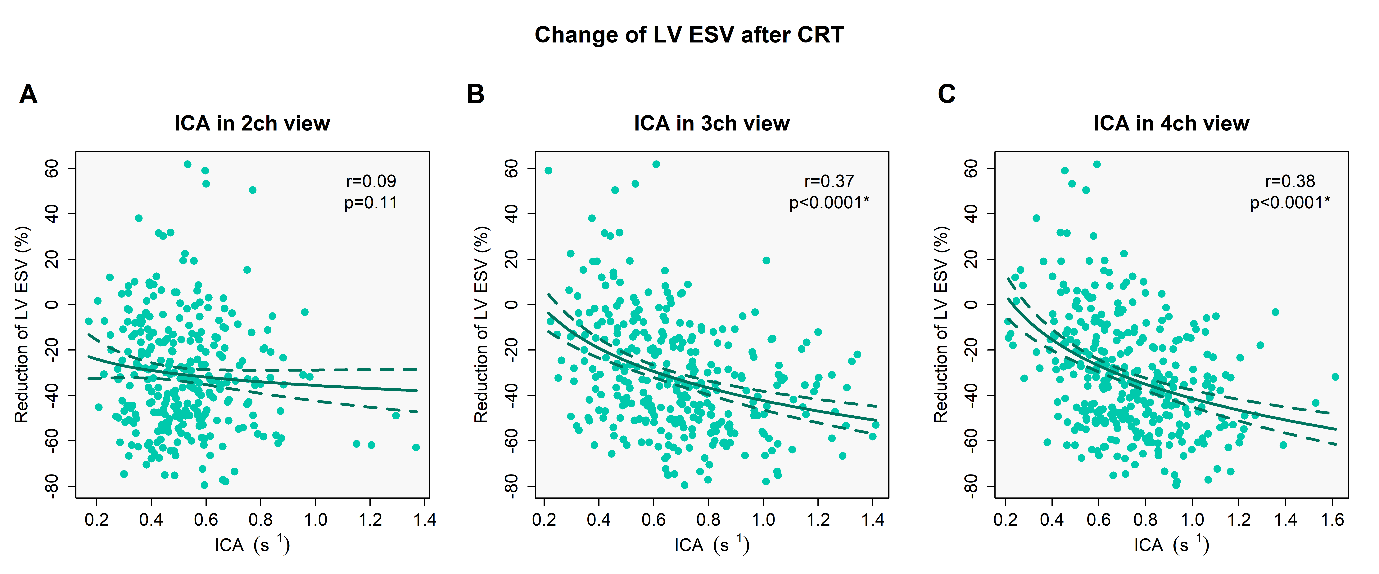


Panels A-C show change of LV ESV after CRT as a function of baseline ICA in 2ch, 3ch, and 4ch views, respectively.

CRT: cardiac resynchronization therapy; ESV: end-systolic volume; ICA: Index of contractile asymmetry; LV: left ventricle; *: p<0.05.
