# Supplementary material for: Contractile asymmetry and survival in patients with left bundle branch abnormality treated with cardiac resynchronization therapy
Source: Eur Heart J Imaging Methods Pract. 2023 Dec 20;1(2):qyad045. doi: 10.1093/ehjimp/qyad045 (PMC11195769; doi:10.1093/ehjimp/qyad045)
Supplement: qyad045_Supplementary_Data [file qyad045_Supplementary_Data.zip › Supplement 1 Ischemic and non-ischemic.docx]

**Supplement 1**

**Index of contractile asymmetry and survival after CRT in patients with *non-ischemic cardiomyopathy*.**


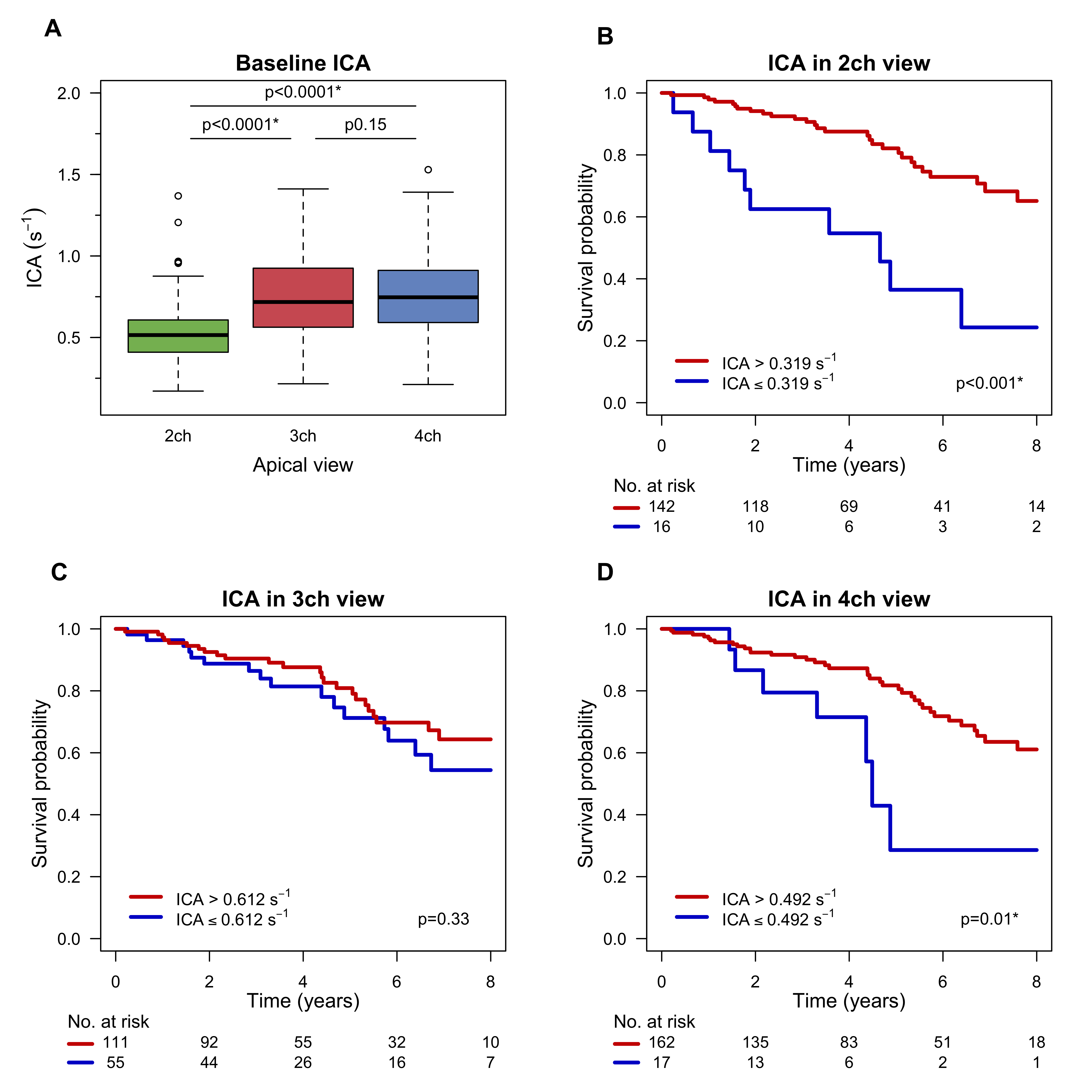


Panel A: boxplots of baseline ICA in all three apical echocardiographic views. Panels B-D: Kaplan-Meier curves for event-free survival based on ICA. High ICA-values are represented by a red curve, and low ICA-values are represented by a blue curve.

ICA: Index of contractile asymmetry; *: p<0.05.

**Univariate survival analysis in patients with *non-ischemic cardiomyopathy*.**

**
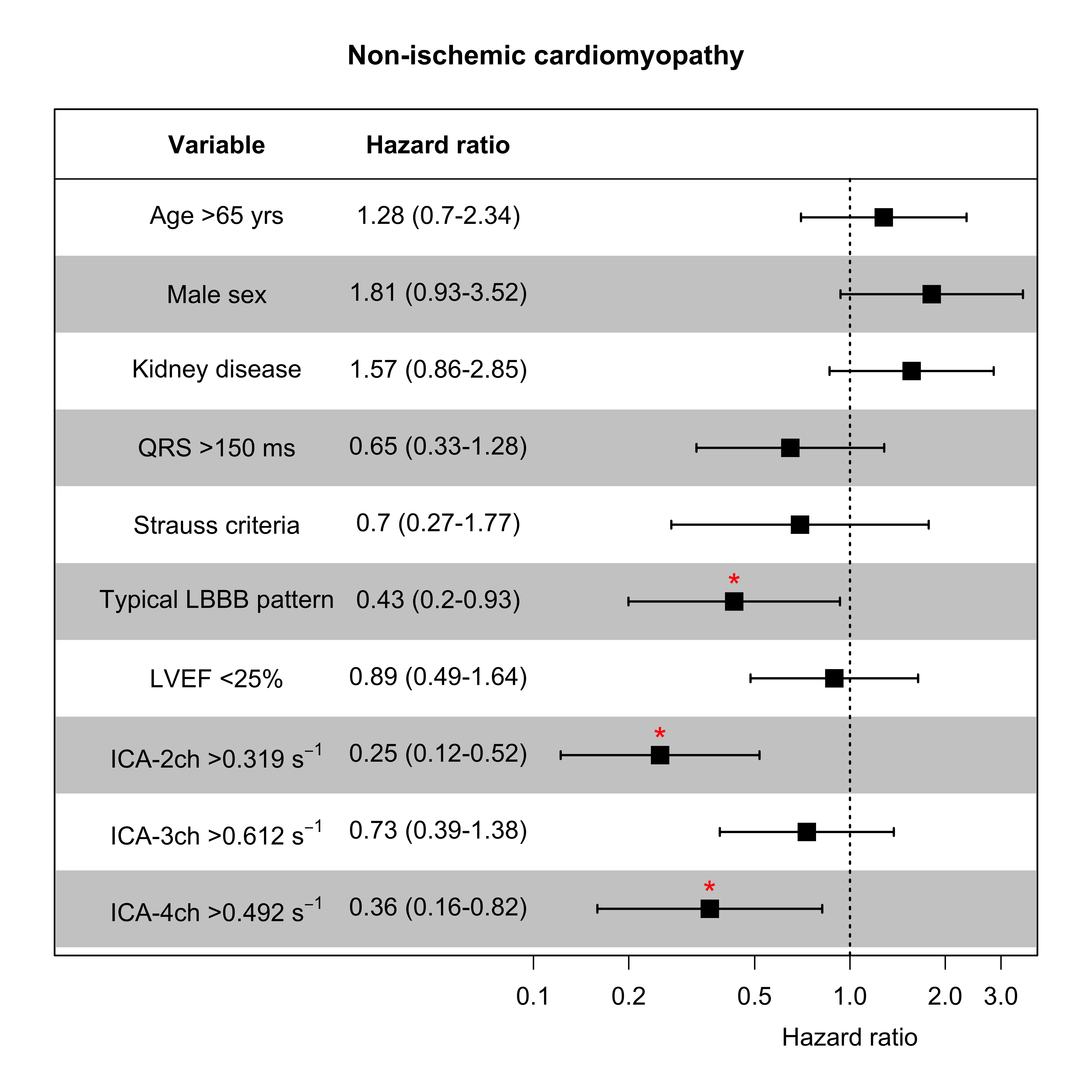
**

CKD: chronic kidney disease; ICA: Index of contractile asymmetry; LBBB: left bundle branch block; LVEF: left ventricular ejection fraction; *: p<0.05.

**Index of contractile asymmetry and survival after CRT in patients with *ischemic*** ***cardiomyopathy*.**


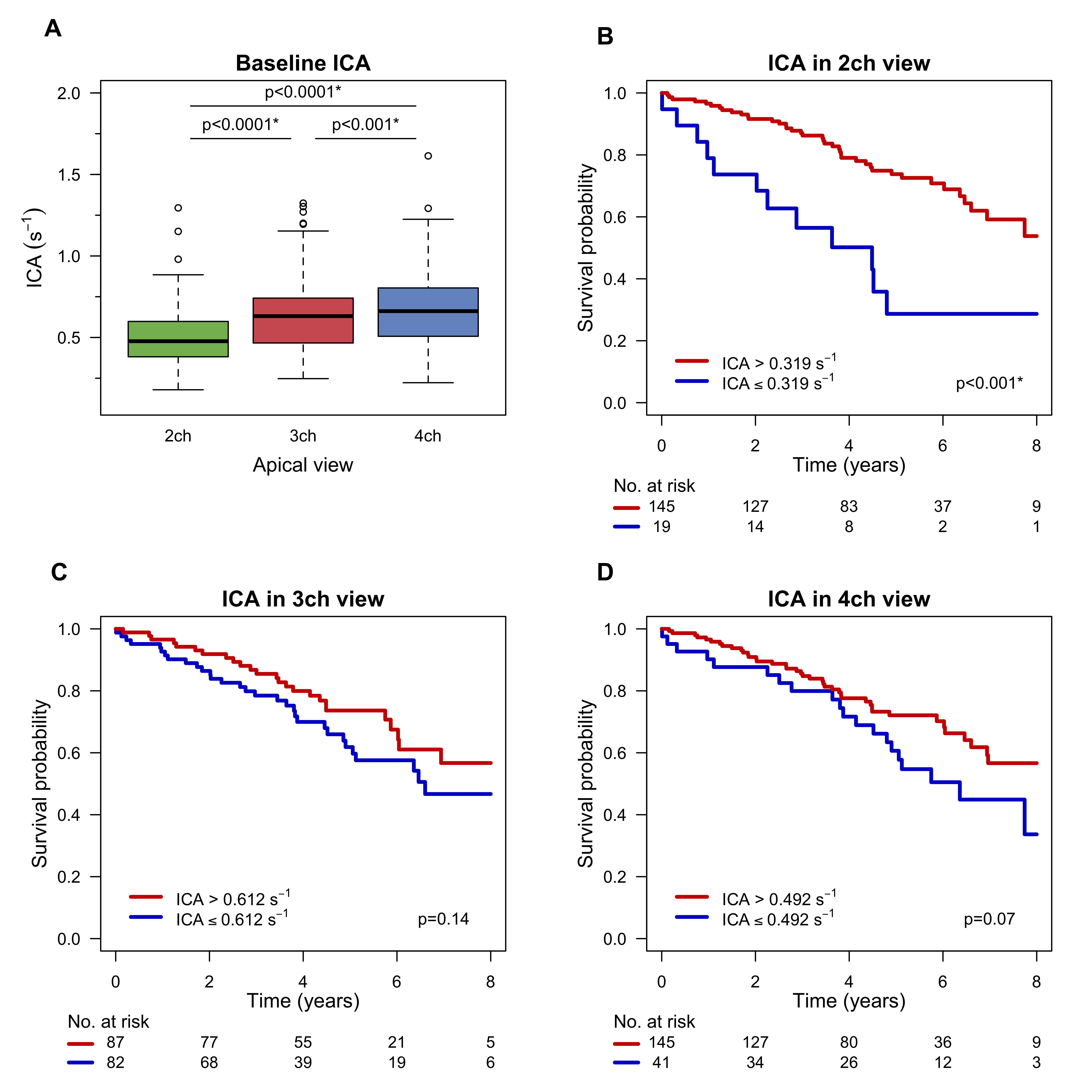


Panel A: boxplots of baseline ICA in all three apical echocardiographic views. Panels B-D: Kaplan-Meier curves for event-free survival based on ICA. High ICA-values are represented by a red curve, and low ICA-values are represented by a blue curve.

ICA: Index of contractile asymmetry; *: p<0.05.

**Univariate survival analysis in patients with *ischemic cardiomyopathy*.**

**
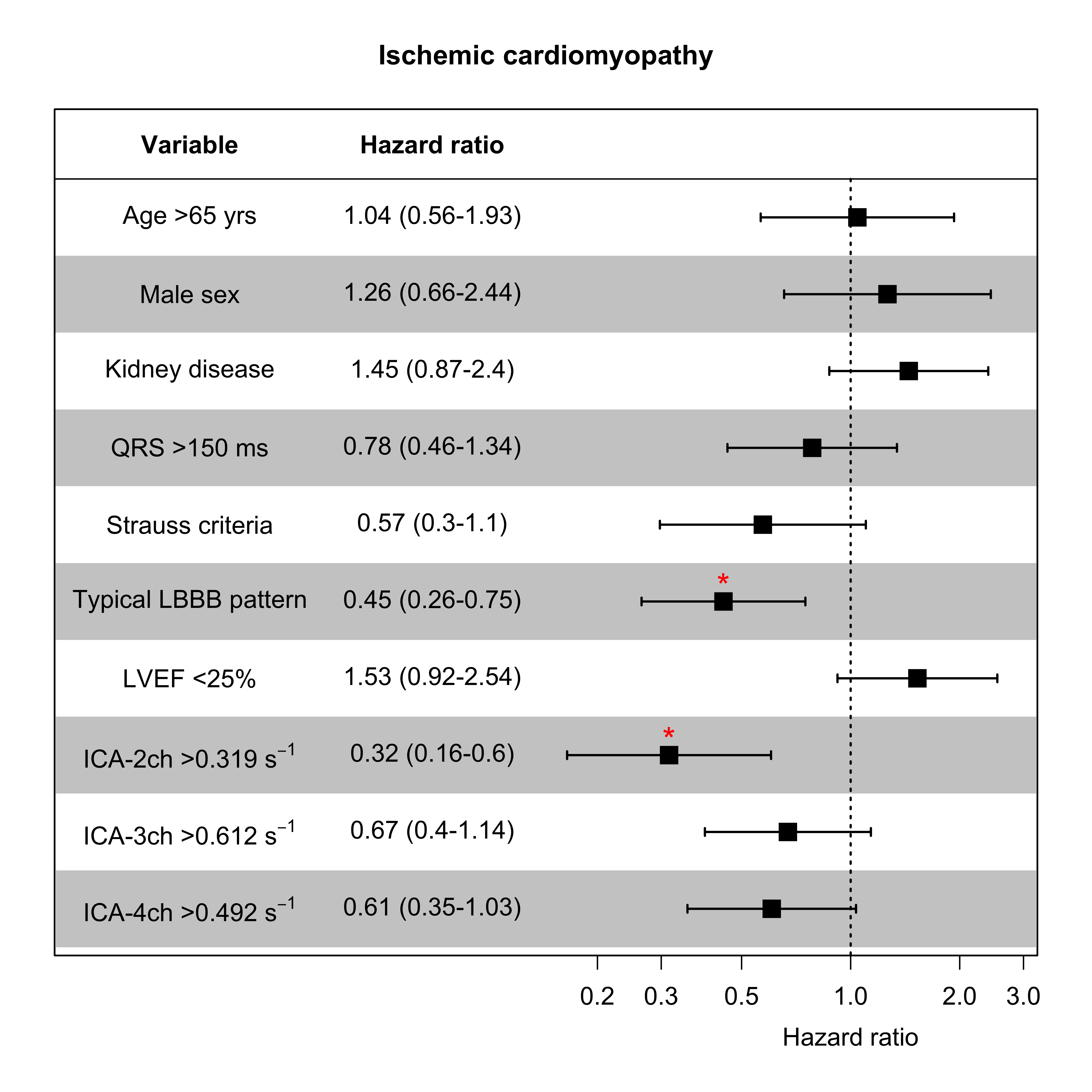
**

CKD: chronic kidney disease; ICA: Index of contractile asymmetry; LBBB: left bundle branch block; LVEF: left ventricular ejection fraction; *: p<0.05.
